# Data of heavy metals in soil and groundwater at Kiwi gardens of Amlash in Guilan Province, Iran

**DOI:** 10.1016/j.dib.2018.04.046

**Published:** 2018-04-21

**Authors:** Dariush Naghipour, Seyed Davoud Ashrafi, Kamran Taghavi

**Affiliations:** a School of Health, Guilan University of Medical Sciences, Rasht, Iran; b Research Center of Health and Environment, Guilan University of Medical Sciences, Rasht, Iran

**Keywords:** Heavy metals, Chemical fertilizer, Soil & groundwater pollution, Amlash

## Abstract

Data on this paper describe the concentrations of arsenic, cadmium, copper, nickel, lead and zinc in the surface soils and groundwater's of Kiwi gardens and its relation to chemical fertilizers in Amlash city, Guilan Province, in Iran. The results of this study showed that the average concentration of heavy metals in groundwater and soils of the studied areas was less than the national standards of Iran for irrigation water, Dutch MPA for soils (except Cu and Ni) and Canadian MAC for inorganic fertilizers. Considering that after fertilizing to soils used in gardening, the concentration of heavy metals in groundwater and soil can be increased significantly, so that chemical fertilizers can be considered as an effective factor in increasing the amount of heavy metals in water and soil. The results of this research can be used by who concern about water and soil quality related to fertilizing and also can be used by Rural Water and Wastewater Company and Ministry of Jahad Agriculture of Iran.

**Specifications table**

**Table:** 

**Subject area**	Environmental Sciences
**More specific subject area**	Heavy metals in ground water soil and fertilizer
**Type of data**	Figure and table
**How data was acquired**	As, Cd, Cu, Ni, Pb and Zn measurements were carried out by ICP-OES – Spectro (Model ARCOS FHE12,Germany) based on standard procedures [1]. Samples of this study were prepared and analyzed from rural ground waters, soils and fertilizers.
	Digital pH meter (Metrohm) was applied for pH regulation after digestion for sample preparations.
**Data format**	Raw, analyzed.
**Experimental factors**	The data were obtained in two season, spring and summer, and pH was measured in field, and for heavy metals analyzing the samples in poly- ethylene bottles were stored in a dark place at 4 °C temperature until the analysis [1].
**Experimental features**	As, Cd, Cu, Ni, Pb and Zn were determined and compared with Iranian standards for irrigation water, Dutch MPA for soils and Canadian MAC for inorganic fertilizers [2], [3], [4].
**Data source location**	Amlash, Guilan Province, Iran.
**Data accessibility**	The data are available within this paper.

**Value of the data**

• The results obtained from this research can be used by the Food and Drug Administration, Iran's Gardening Department.
• The results of this research can be used by Rural Water and Wastewater Company and Ministry of Jahad Agriculture of Iran.
• The results of this research can be used by the Iranian Environmental Protection Agency, Ministry of Health for human health risk assessment of Gardening products.

## 1 Data

Environmental pollution like drinking water and soil pollution by organic and inorganic materials is one of the most important issues in the world [5], [6], [7], [8], [9], [10], [11], [12], [13], [14], [15], [16]. Before fertilizing the fruit gardens, average concentration of Cd, Cu, Ni, Pb and Zn in groundwater samples was 0.185, 0.579, 3.407, 0.238 and 2.490 µg/l, respectively, but, after the fertilization the mean concentration increased to 0.216, 0.717, 5.435, 0.435 and 4.652 µg/l, respectively. It should be noted that the amount of As in groundwater samples was not detectable due to the accuracy of the device. The average pH of well water in the samples was 6.9. The concentration of heavy metals in groundwater samples before and after fertilization is presented in Table 1. The concentration of heavy metals studied in well water samples, in both sampling stages was lower than the national standards of Iran for irrigation [2].

**Table 1:** Heavy metal concentration in well water samples before and after fertilization in Kiwi Gardens, Amlash City in 2017(µg/l).

Number of samples	**As**		**Cd**		**Cu**		**Ni**		**Pb**		**Zn**	
	before	after	before	after	before	after	before	after	before	after	before	after
1	ND	ND	0.172	0.193	0.459	0.653	2.510	8.670	0.362	0.759	7.170	7.480
2	ND	ND	0.129	0.159	0.699	1.320	2.200	9.400	ND	0.257	5.201	6.050
3	ND	ND	0.160	0.169	ND	1.810	3.630	4.310	0.192	0.408	2.480	6.190
4	ND	ND	0.180	0.194	ND	1.390	2.520	4.970	ND	0.430	1.760	5.320
5	ND	ND	0.153	0.361	ND	1.950	2.410	4.570	0.282	0.379	3.970	9.520
6	ND	ND	0.135	0.223	ND	0.048	3.790	5.830	0.227	0.313	2.150	3.070
7	ND	ND	0.133	0.183	ND	0.486	2.880	4.070	0.282	0.422	3.160	3.200
8	ND	ND	0.138	0.144	ND	0.173	5.180	5.300	0.310	0.361	2.080	2.800
9	ND	ND	0.113	0.235	ND	0.043	7.510	8.610	0.220	0.226	1.310	2.150
10	ND	ND	0.163	0.169	ND	0.109	5.230	6.220	0.180	0.320	0.999	2.430
11	ND	ND	0.194	0.243	ND	0.474	3.550	4.610	0.249	1.02.	0.880	2.530
12	ND	ND	0.153	0.324	ND	ND	5.990	7.010	ND	0.357	1.470	3.870
13	ND	ND	0.111	0.226	ND	ND	3.790	5.500	0.226	0.280	1.890	4.560
14	ND	ND	0.960	0.171	ND	1.290	2.620	4.090	0.156	0.225	1.05.	1.78
15	ND	ND	0.102	0.229	ND	0.534	1.080	4.100	0.241	0.393	1.270	2.850
16	ND	ND	0.119	0.206	ND	ND	1.720	3.030	0.240	0.270	1.930	2.220
17	ND	ND	0.076	0.275	ND	ND	2.850	4.170	0.112	0.755	1.310	2.570
18	ND	ND	0.164	0.165	ND	ND	4.430	5.620	0.281	0.287	4.260	6.410
19	ND	ND	0.143	0.156	ND	0.104	2.970	5.180	0.244	0.259	1.930	7.74
20	ND	ND	0.202	0.296	ND	0.374	1.280	3.440	0.251	0.982	3.540	10.300
Average	ND	ND	0.185	0.216	0.579	0.717	3.407	5.435	0.238	0.435	2.490	4.652
Min	ND	ND	0.076	0.144	0.459	0.043	1.080	3.030	0.112	0.225	0.880	1.780
Max	ND	ND	0. 960	0.361	0.699	1.950	7.510	9.400	0.362	1.020	7.170	10.300
S.D	ND	ND	0.185	0.059	0.169	0.658	1.615	1.767	0.058	0.242	1.624	2.586
Iranian Irrigation Standards	1		10		200		200		5000		2000	

Before the fertilization, the mean concentration of As, Cd, Cu, Ni, Pb and Zn in soil samples was 0.517, 0.066, 18.386, 10.151, 13.091 and 39.745 mg/kg, respectively, but, after the fertilization the mean concentration was 0.273, 0.085, 25.048, 14.555, 16.226 and 52.186 mg/kg, respectively. The concentration of heavy metals studied in soil samples in both sampling stages was lower than the Dutch MPA for soils [3]. According to the soil texture analysis in the region, the average soil pH was 6.5. The concentration of metals in soil samples before and after fertilization is presented in Table 2.

**Table 2:** Heavy metal concentrations in soil samples before and after fertilization in Kiwi Gardens, Amlash City in 2017 (mg/kg).

Number of samples	As		Cd		Cu		Ni		Pb		Zn	
	before	after	before	after	before	after	before	after	before	after	before	after
1	ND	0.680	0.020	0.120	28.580	35.970	8.910	15.710	15.830	18.110	39.890	83.870
2	0.007	ND	ND	ND	17.680	19.730	8.860	10.220	14.960	17.840	32.380	52.540
3	ND	ND	ND	0.050	14.900	37.030	9.890	13.580	12.390	14.100	38.0100	40.600
4	ND	ND	ND	ND	24.660	35.740	6.390	13.880	14.140	15.440	29.300	41.900
5	0.300	ND	ND	0.004	19.050	23.490	9.330	20.850	13.710	15.130	35.920	36.550
6	ND	ND	ND	0.002	23.250	23.400	11.900	11.980	13.110	14.970	32.660	43.600
7	0.090	ND	ND	0.310	8.070	42.25.	16.450	18.020	14.43.	24.520	42.270	60.050
8	0.130	ND	ND	ND	24.580	26.380	12.250	13.090	14.870	15.990	37.530	54.450
9	ND	ND	ND	0.04	20.850	24.410	10.09	13.400	13.720	18.090	47.400	57.840
10	ND	ND	ND	0.001	16.440	24.200	10.600	17.100	14.770	18.940	49.600	69.120
11	ND	ND	ND	ND	17.420	25.710	10.03	14.770	12.080	15.720	24.220	75.460
12	0.140	ND	ND	ND	20.930	23.110	10.290	24.280	13.610	15.900	38.150	47.710
13	ND	ND	ND	ND	21.890	24.850	14.200	16.360	15.610	17.100	37.820	42.630
14	0.780	ND	ND	ND	3.420	14.670	8.270	9.100	15.240	16.800	28.290	30.570
15	0.600	ND	0.130	0.19	14.180	16.410	9.850	12.220	14.320	15.450	31.300	35.630
16	0.500	ND	ND	ND	18.090	20.050	8.900	11.910	11.570	12.010	83.530	87.850
17	ND	ND	ND	ND	22.490	22.990	11.870	16.780	15.800	16.070	43.800	45.020
18	0.72	ND	ND	ND	20.180	21.160	9.500	13.240	13.520	14.350	46.040	60.490
19	1.260	0.090	0.050	0.090	14.03	21.900	7.110	14.430	9.200	14.290	45.510	46.500
20	1.16	0.050	ND	0.050	17.030	17.510	8.330	10.180	13.380	13.700	31.290	31.3500
Average	0.517	0.273	0.066	0.085	18.386	25.048	10.151	14.555	13.091	16.226	39.745	52.186
Min	0.007	0.050	0.020	0.001	3.420	14.670	6.390	9.100	11.570	12.010	24.220	30.570
Max	1.260	0.680	0.130	0.310	28.580	42.250	16.450	24.280	15.830	24.520	83.530	87.850
S.D	0.431	0.352	0.056	0.098	5.790	7.280	2.328	3.668	3.475	2.588	12.409	16.545
The Dutch MPA for soils	1–10		<1		1–10		1–10		>10		>10	

The concentration of heavy metals in five fertilizers used in kiwi gardens is presented in Table 3. The Average concentration of heavy metals in fertilizer samples was lower than the Canadian MAC standards (except for Zn) [4].

**Table 3:** Heavy metals concentrations in five highly used fertilizers of Kiwi Gardens, Amlash City in 2017 (mg/kg).

Parameters	As	Cd^+^	Cu	Ni	Pb	Zn
Urine	0.668	0.027	0.642	12.100	1.220	8.770
Triple superphosphate	9.060	0.013	5.260	9.130	1.650	7.030
Potassium sulfate	0.160	0.078	0.601	13.940	0.420	1570.950
Zinc sulfate	0.118	3.530	0.524	66.050	2.554	28592.300
Complete	1.137	0.047	4.086	13.203	0.420	610.700
Average	2.228	0.739	2.222	22.884	1.252	1017.950
SD	3.841	1.560	2.275	24.199	0.899	1227.319
Min	0.668	0.013	0.524	9.130	0.420	7.030
Max	9.060	3.530	5.260	66.050	2.554	28592.300
Canadian MAC	75	20	–	180	500	1850

Average of five highly used fertilizers of Kiwi Gardens in 7 villages in Amlash is presented in Table 4, and showed that urea, triple super phosphate, potassium sulfate, zinc sulfate and full fertilizer in the Kiwi gardens were 420, 380, 200, 90 and 75 kg/yr.

**Table 4:** Average of five highly used fertilizers of Kiwi Gardens, Amlash City in 2017 (kg/yr.).

**Name of village**	**Urine**	**Triple superphosphate**	**Potassium sulfate**	**Zinc sulfate**	**Full fertilizer**
**Balangheh**	27	32	15	5	15
**Hardoab**	33	28	25	10	5
**Tarkeh**	45	42	20	12	17
**Azarin**	64	58	35	10	10
**Lagmoj**	73	64	28	13	8
**Holosara**	80	73	32	20	10
**Chamanestan**	98	83	45	20	10
**Total**	420	380	200	90	75

## 2 Experimental design, materials and methods

### 2.1 Study area description

**Fig. 1:**
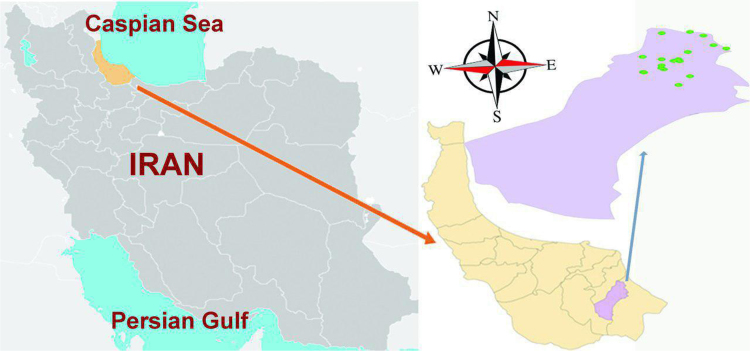
Sampling zone and point of research, Amlash, Guilan Province.

The study area is located in the east of Giulan, Amlash city, 75 km from Rasht, the center of the province. According to the last census of Statistical Center of Iran at 2017 the population of the city of Amlash has been declared to be 43,225 people. The studied area is under cultivation of fruit gardens, which is about 20 ha. These gardens are located in 7 villages called Chamanestan, Nerke, Hardoab, Legmog, Balange, Holosara and Azarin. Sampling zone and point of this research was presented in Fig. 1.

### 2.2 Sample collection and analytical procedures

Samples collected at 21 stations from wells and soils of Kiwi gardens before and after adding fertilizers (Urea, triple superphosphate, potassium sulfate, zinc sulfate and full fertilizer). Data obtained with two methods which were questionnaire and analyzing.Questionnaires were provided to farmers in order to obtain information about the type, amount, manner of fertilizer use, and area of cultivation for kiwi gardens. The distribution of fertilizers was collected from the Jahad Agricultural Organization and the fertilizer distribution cooperatives, and the fertilizers were purchased from its supply stores. In water sampling of wells as a source for irrigation of the farms, samples took from well discharge. However, in fields where their irrigation water were supplied from various sources, such as springs and rivers, especially during well dehydration, the samples were prepared in a composite form. After filtering well water (with Whatman filter 42) and condensation process at 90 °C in polyethylene bottles, samples labeled. Then, with 1 ml of concentrated nitric acid, pH of samples decreased to less than 2. The samples were transferred to the laboratory using ice bag and kept in the refrigerator [1]. In soil sampling, samples were collected at the stations designated by the combined method. Samples of surface soils were prepared at a depth of 0–20 cm and mixed together and shaking in the laboratory for one hour. Soil samples were mixed with distilled water at a rate of 2.5:1 in the laboratory. Then the pH of the mixture was measured using pH meter. Preparation of fertilizer samples was done as well as soil samples [17].

To prepare a fertilizer sample, 5 fertilizers were selected for harvesting fruit gardens and from each kind of fertilizer 3 different brands were prepared on the market and each brand was harvested with equal ratio and mixed together and finally 5 fertilizer samples (sample weight; 1 Kg), were prepared and tested. Digestion of soil and fertilizer samples were carried out using nitric acid and concentrated hydrochloric acid of Merck Germany and deionized distilled water. Heavy metals of samples were digested with a mixture of three parts of hydrochloric acid and one nitric acid fraction. All samples, prepared, digested and measured according to standard methods [1], [18].

## Funding sources

This paper was a part of faculty approved research project and supported financially by a grant (No: 93122606) from Guilan University of Medical Sciences, Rasht, Iran.
