# Supplementary material for: Data of heavy metals in soil and groundwater at Kiwi gardens of Amlash in Guilan Province, Iran
Source: Data Brief. 2018 Apr 21;18:1556–61. doi: 10.1016/j.dib.2018.04.046 (PMC5999521; doi:10.1016/j.dib.2018.04.046)
Supplement: Supplementary file 1 — Supplementary material [file mmc1.docx]

**Conflict of interest**

All author confirms no conflict of Interset.
